# Eligibility Criteria of Randomized Clinical Trials in Critical Care Medicine

**DOI:** 10.1001/jamanetworkopen.2024.54944

**Published:** 2025-01-17

**Authors:** Alya Heirali, Kiyan Heybati, Jariya Sereeyotin, Faizan Khan, Christopher Yarnell, Karla Krewulak, Srinivas Murthy, Karen E. A. Burns, Robert Fowler, Kirsten Fiest, Sangeeta Mehta

**Affiliations:** 1 Department of Critical Care Medicine, University of Calgary, Calgary, Alberta, Canada; 2 Alix School of Medicine, Mayo Clinic, Rochester, Minnesota; 3 Department of Anesthesiology, Division of Critical Care Medicine, King Chulalongkorn Memorial Hospital and Faculty of Medicine, Chulalongkorn University, Bangkok, Thailand; 4 Department of Clinical Neurosciences and Hotchkiss Brain Institute, University of Calgary, Calgary, Alberta, Canada; 5 Interdepartmental Division of Critical Care Medicine, University of Toronto, Toronto, Ontario, Canada; 6 Department of Critical Care Medicine, Scarborough Health Network, Toronto, Ontario, Canada; 7 Department of Pediatrics, Faculty of Medicine, University of British Columbia, Vancouver, British Columbia, Canada; 8 Li Ka Shing Knowledge Institute, St Michael’s Hospital, Toronto, Ontario, Canada; 9 Sunnybrook Health Sciences Centre, Toronto, Ontario, Canada; 10 Department of Medicine, Sinai Health System, Toronto, Ontario, Canada

## Abstract

**Importance:**

Eligibility criteria for randomized clinical trials (RCTs) are designed to select clinically relevant patient populations. However, not all eligibility criteria are strongly justified, potentially excluding marginalized groups, and limiting the generalizability of trial findings.

**Objective:**

To summarize and evaluate the justification of exclusion criteria in published RCTs in critical care medicine.

**Evidence Review:**

A systematic sampling review of parallel-group RCTs published in the top 5 general internal medicine journals by impact factor (*The Lancet, New England Journal of Medicine, Journal of the American Medical Association, British Medical Journal, *and *Annals of Internal Medicine*) between January 1, 2018, and February 23, 2023, was conducted. RCTs enrolling adults in intensive care units (ICUs) and RCTs enrolling critically ill patients who required life-sustaining interventions typically initiated in the ICU were included. All study exclusion criteria were categorized as either poorly justified, potentially justified, or strongly justified, adapting previously established criteria, independently and in duplicate.

**Findings:**

In total, 225 studies were identified, 75 of which were included. The median (IQR) number of exclusion criteria per trial was 19 (14-24), with 1455 total exclusion criteria. Common exclusion criteria were related to the risk of adverse reaction to interventions (302 criteria [20.8%]), followed by inability to obtain consent (120 criteria [8.2%]), and treatment limitation decisions (97 criteria [6.7%]). Most exclusion criteria were either strongly justified (1080 criteria [74.2%]) or potentially justified (297 criteria [20.4%]), whereas 5.4% (78 criteria) were poorly justified. Of the 78 poorly justified exclusion criteria, the most common were pregnancy (19 criteria [24.4%]), communication barriers (11 criteria [14.1%]), lactation (10 criteria [12.8%]), and lack of health insurance (10 criteria [12.8%]). Overall, 45 of 75 studies (60.0%) had at least 1 poorly justified exclusion criteria.

**Conclusions and Relevance:**

Most exclusion criteria in critical care medicine RCTs were strongly justifiable. Across poorly justified criteria, the most common exclusions were pregnant or lactating persons, those with communication barriers, and individuals without health insurance. This highlights the need to carefully consider exclusion criteria when designing trials to minimize the inappropriate exclusion of participants and enhance generalizability.

## Key Points

**Question:**

Are exclusion criteria in critical care randomized clinical trials (RCTs) published in *The Lancet*, *British Medical Journal*, *Journal of the American Medical Association*, *New England Journal of Medicine*, and *Annals of Internal Medicine* strongly justifiable, potentially justifiable, or poorly justifiable?

**Findings:**

In this systematic sampling review of 75 critical care RCTs published in high-impact medical journals, 94.6% of exclusion criteria were strongly justifiable or potentially justifiable. However, 60.0% of trials included at least 1 poorly justified exclusion criterion, with the most common being pregnancy, communication barriers, lactation, and lack of health insurance.

**Meaning:**

This review highlights the need for rigorous evaluation of exclusion criteria in study design, which will limit poorly justified exclusion of potential trial participants and improve generalizability of trial findings.

## Teaser

This study examines the justification of exclusion criteria in published randomized clinical trials in critical care medicine.

## Introduction

Randomized clinical trials (RCTs) are the criterion standard in medical research, and their findings have the potential to impact patient care around the world.^1^ Although eligibility criteria play a critical role in selecting at-risk patient populations and reducing confounding, overly stringent exclusion criteria may limit the reproducibility of trial findings, leading to a lack of generalizability. This issue may stem from limitations in study design, limited diversity and representation in research teams, and the need for patient and community engagement. These limitations in the design of RCTs may result in the exclusion of patients on the basis of age, sex, and disability.^2,3^

In a systematic sampling review^3^ of eligibility criteria of RCTs published in high-impact medical journals between 1994 and 2006, trial participants were most often excluded on the basis of age, sex, comorbidities, cotreatment, socioeconomic status, communication barriers, participation in other trials, ethnicity, and inability to provide consent. However, there are limited data examining the justification of exclusion criteria across clinical trials in critical care medicine. Critical care patients present with unique health conditions compared with patients in other medical specialties. Understanding the specific exclusion criteria used in critical care RCTs ensures that the findings of these trials are relevant and applicable to this patient population.^4^ By systematically reviewing exclusion criteria in critical care RCTs and their justification, researchers can address gaps in knowledge and critically examine the validity of proposed exclusion criteria during study design. Accordingly, we performed a review of RCTs of adults with critical illness to catalog exclusion criteria and to evaluate the justifications for those exclusion criteria. We also summarized author demographics because diverse research teams may support more inclusive eligibility criteria. We hypothesized that poorly justified exclusion criteria may be prevalent in RCTs enrolling critically ill adult patients. Identification of poorly justified exclusion criteria in critical care RCTs may encourage investigators to rigorously appraise exclusion criteria, thereby reducing bias, enhancing external validity, and maximizing the benefits of interventions.

## Methods

### Data Sources and Eligibility Criteria

In this systematic sampling review, we used previously established search criteria to identify critical care RCTs^5,6^ published in English between January 1, 2018, and February 23, 2023, in the top 5 impact factor general medical journals: *The Lancet, New England Journal of Medicine (NEJM)*, *Journal of the American Medical Association (JAMA)*, *British Medical Journal (BMJ)*, and *Annals of Internal Medicine*.^7,8^ The rationale for limiting our review to these journals is that they likely have the most stringent diversity reporting requirements, as well as the greatest impact on patient care. We included parallel-group RCTs that enrolled adult patients who were primarily (≥50%) treated in general medical intensive care units (ICUs), trauma ICUs, burn ICUs, surgical ICUs, or neurological ICUs. Trials consenting and/or enrolling critically ill participants, defined as those receiving life-sustaining interventions (eg, invasive mechanical ventilation) usually started in the ICU, but may also be used in other settings (eg, emergency department, acute care wards, or intermediate care units) were included if the majority (≥50%) of patients were recruited and/or admitted to an ICU setting.

Trials were included if they enrolled patients after cardiac arrest who were treated in general medical ICUs, trauma ICUs, burn ICUs, or neurological ICUs. However, trials enrolling patients from coronary or cardiovascular ICUs were excluded, owing to the unique characteristics of these patient populations, generally comprising individuals recovering from cardiac surgery. In addition, the following publications were excluded: nonoriginal research articles, retracted publications, RCTs focusing on nonpatient participants such as clinicians, secondary or post hoc analyses of published trials, pilot and vanguard studies, and cluster or crossover trials. This study did not require ethics approval or informed consent because publicly available data were used and no human participants were involved, in accordance with 45 CFR §46.

### Definitions

We conducted a review of 5 high-impact internal medicine journals. As described previously by Van Spall et al,^3^ it was necessary to establish what defines an eligibility criterion. A trial’s inclusion criteria described the medical condition as well as criteria for entry or recruitment of participants.^3^ All other eligibility criteria were defined as exclusion criteria.^3^ Exclusion criteria were assessed as poorly justified, potentially justified, or strongly justified by adapting previously established guidelines by 2 authors (A.H. and K.H.) independently and in duplicate, with disagreements resolved through discussion (Box).^3^ Exclusion criteria were defined as strongly justified if there was a clinical reason for exclusion, such as if there was a risk to patient safety due to anaphylaxis, known contraindication in states such as kidney failure or hepatic dysfunction, or the patient was moribund with goals of care that were not consistent with intervention. Poorly justified exclusion criteria included any criterion that was not strongly justifiable according to existing studies and/or drug manufacturer instructions and typically included individual patient characteristics such as age, sex-specific conditions (eg, pregnancy, fertility, or lactation), cognitive dysfunction, and language barriers, among others. Potentially justified exclusion criteria were neither strongly nor poorly justified, such as the opinion of the physician and/or clinical care team. Vaguely defined exclusion criteria (ie, “other unspecified reasons”) could not be accurately assessed and were, therefore, excluded from the analyses. The discrepancy between the total number of exclusion criteria and studies arises from instances where individual exclusion criteria are counted separately within a single study, even though they contribute to the same overarching exclusion criteria. This occurs when multiple criteria address different aspects of an overarching reason for exclusion. Therefore, although the total number of studies remains constant, the total number of exclusion criteria may exceed the number of studies because of these duplications.

Box. **Definitions for Justification of Exclusion Criteria^a^**

**Strongly Justified Reason for Excluding Individuals From a Critical Care RCT**

Individual or substitute decision-maker is unable to grant informed consent
Intervention or placebo would likely be harmful
  Unacceptable risk of known adverse reaction to intervention
  Unacceptable risk of assignment to placebo or withholding of intervention
Intervention would likely be ineffective
  Individual not likely to have condition of interest
  Individual not at risk for outcome
  Individual has type of disease that is likely not to respond to treatment
Effect of intervention will be difficult to interpret
  Individual has a cointervention that will likely confound the treatment effect
  Individual has an independent condition with signs and symptoms similar to the condition of interest that will make the treatment effect difficult to assess (eg, allergic rhinitis and upper respiratory tract infections)

**Poorly Justified Reason for Excluding Individuals From a Critical Care RCT**

Is not a strongly justifiable reason as described above
The exclusion is based on 1 or more of the following factors^b^: age; sex; sex-specific conditions such as menstruation, pregnancy, fertility or lactation; sexual orientation; gender; racial, ethnic, or religious background; spoken or written language ability; educational background; socioeconomic status; employment status; marital status; cognitive ability, mental impairment, or intelligence quotient; physical ability or disability; chronic health conditions; comorbidity; housing (eg, individuals experiencing homelessness or precarious housing); or geographic location (eg, too far from medical or research center)
The condition under investigation and or intervention is not specific to the factors listed above
The factors described above have no direct bearing on the condition, intervention, or results

**Potentially Justified Reason for Excluding Individuals From a Critical Care RCT**

Is neither a strongly justified nor poorly justified reason as described above
Individual may not adhere to intervention
Individual may not complete follow-up
Opinion of physician
Short life expectancy
Participation in other trial
Concomitant medication

Abbreviation: RCT, randomized clinical trial.

^a^
Adapted from Van Spall et al.^3^

^b^
This is not a comprehensive list of all possible individual factors used as exclusion criteria.

### Trial Selection

Two authors (A.H. and K.H.) reviewed titles and abstracts independently and in duplicate using Covidence systematic review software (Veritas Health Innovation) to select articles that met inclusion criteria. Articles that did not meet the eligibility criteria were excluded. Full texts of the articles were subsequently reviewed to identify eligible studies. Disagreements were resolved by discussion until consensus was reached. If reviewers were unable to reach consensus, a third author (S.M.) helped with adjudication.

### Trial Characteristics

Study characteristics, inclusion and exclusion criteria, and justification for each exclusion criterion were extracted independently, in duplicate. Justifications were defined as examples, references, and or definitions provided by authors to explain specific exclusion criteria including those noted in supplementary materials. The criteria were then assessed as poorly justified, strongly justified, or potentially justified in a similar manner, using previously established definitions (Box),^3^ and independent of any investigator-provided justifications. Where discordance was present between extractors regarding the assessment of exclusion criteria, discussion occurred until consensus was reached. If consensus was not reached, additional reviewers resolved conflicts.

Data were extracted from manuscript main texts, figures, tables, supplemental content, and published protocols. Trial registry websites (eg, ClinicalTrials.gov) were used to extract study accrual start and end dates and/or trial phase if this information was not listed in the main text or supplements. The corresponding author was contacted if extractors needed further clarifications. Extracted study characteristics included publication title and year; DOI (digital object identifier); journal source; first, last, and corresponding authors’ full name; first and last author sex, race, and geographic location^8^; funding sources (ie, public agency [government or hospital grant], private [industry], mixed [public and private], in-kind support, unfunded, or unclear); type of intervention (ie, drug, ventilation strategy, protocol, surgery, device, or other); single-center or multicenter conduct; number of recruiting sites; geographic scope (ie, countries and whether they were low or middle-income countries [LMICs])^9^; study accrual start and end dates; blinding (ie, none, single, or double); placebo-controlled, standard of care controlled, controlled with other intervention, or controlled with no intervention; trial phase (ie, 1, 2, 3, or 4); and number of individuals screened for eligibility, excluded, and randomized. Positive trials were defined as trials that demonstrated a statistically significant (*P* < .05) difference between the intervention and the controls for at least 1 primary end point.

### Author Demographics

For first and last author demographics, we used previously described methods to attribute sex and race.^10,11^ Sex was ascribed as follows: (1) the author was personally known to 1 of the investigators; (2) internet searches of individual authors for pronouns or photographs; and (3) use of NamSor (NamSor SAS), which identifies a person’s sex with 97% accuracy based on first name. We recorded whether the first and last authors belonged to racial or ethnic minority groups, adapting the definition established by the Canadian Employment Equity Act as, “persons, other than Aboriginal peoples, who are non-Caucasian in race, or non-White in colour.”^10^ Whether a person belonged to a racial or ethnic minority group was determined as follows: (1) the author is personally known to 1 of the investigators; and (2) internet searches of individual authors for photos or reference to race or ethnicity.

### Statistical Analysis

Descriptive statistics were used to report trial characteristics, author demographics, number of inclusion and exclusion criteria, reported justification of eligibility criteria, and assessment of exclusion criteria as poorly, potentially, or strongly justified. Continuous variables were presented as median (IQR) given the nonnormal distributions. Analyses were conducted in R statistical software version 4.2.1 (R Project for Statistical Computing) using the R Studio 2022.02 integrated development environment (PBC). Risk of bias assessments was not done as we did not assess trial outcomes but rather reported and assessed justification of exclusion criteria. We did not conduct a meta-analysis given the heterogeneity among RCTs.

## Results

### Trial Characteristics

A total of 225 studies were identified using our search strategy, 75 of which met eligibility criteria (Figure).^12,13,14,15,16,17,18,19,20,21,22,23,24,25,26,27,28,29,30,31,32,33,34,35,36,37,38,39,40,41,42,43,44,45,46,47,48,49,50,51,52,53,54,55,56,57,58,59,60,61,62,63,64,65,66,67,68,69,70,71,72,73,74,75,76,77,78,79,80,81,82,83,84,85,86^ Most of the studies were published in *JAMA *(41 studies [54.7%]) and *NEJM* (28 studies [37.3%]) (eAppendix and eTable 1 in Supplement 1). The majority of trials were multicenter (74 studies [98.7%]) and multinational, including participants from 3 or more countries (26 studies [34.7%]) (eTable 1 in Supplement 1). Patients were enrolled from 46 unique countries; the majority of which were high-income countries (32 of 46 countries [69.6%]), and only 1 study (2.2%) enrolled patients from a low-income country (eTable 1 in Supplement 1). Most of the 75 studies were phase 3 (29 studies [38.7%]), open-label (41 studies [54.7%]), drug trials (33 studies [44.0%]), and compared interventions with standard of care (42 studies [56.0%]) (eTable 1 in Supplement 1). The majority of trials were publicly funded (59 studies [78.7%]) (eTable 1 in Supplement 1). The most common conditions of interest were respiratory ones (21 studies [28.0%]), followed by sepsis (14 studies [18.7%]) (eTable 1 in Supplement 1).

**Figure.**
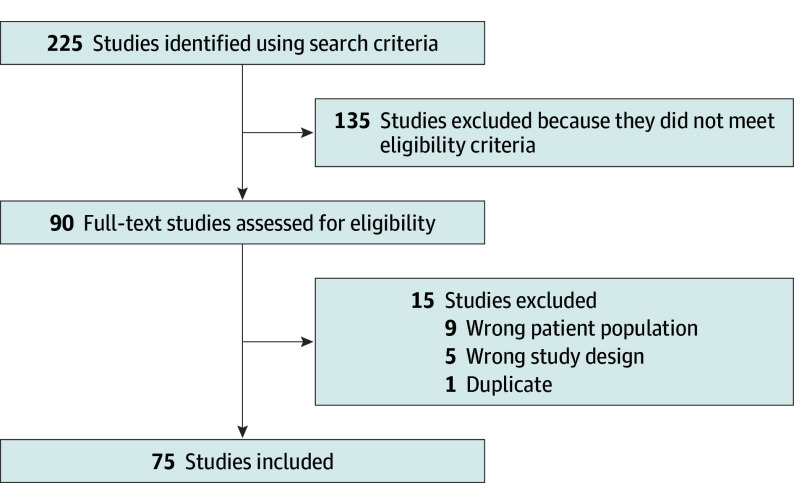
Flowchart of Included Studies

### Author Demographics

The majority of first authors were identified as White (60 studies [80.0%]) and male (61 studies [81.3%]) (eTable 2 in Supplement 1). Similarly, most last authors were identified as White (62 studies [82.7%]) and male (62 studies [82.7%]) (eTable 2 in Supplement 1). Only 20.0% (15 studies) of first and 17.3% (13 studies) of last authors were identified as members of a racial or ethnic minority group (eTable 2 in Supplement 1); none were Black. The largest proportion of first and last authors were located in France (34 of 150 authors [19.4%]) and the US (23 of 150 authors [15.3%]) (eTable 2 in Supplement 1).

### Summary of Exclusion Criteria

In total, 1479 unique exclusion criteria were identified across the 75 RCTs (eTable 3 in Supplement 1); 24 (1.6%) were vaguely defined as other unspecified reasons and, therefore, were excluded from analyses, leaving 1455 exclusion criteria for analysis. The median (IQR) number of exclusion criteria per study was 19 (14-24) criteria (range, 7-43 criteria). The majority of studies excluded patients due to unacceptable risk of known adverse reaction to the intervention (71 of 75 studies [94.7%]), accounting for 20.8% (302 of 1455 criteria) of all exclusion criteria (eTable 3 in Supplement 1). Patient and/or substitute decision-maker not providing informed consent was an exclusion criterion in 85.3% of studies (64 studies); this category accounted for a total of 8.2% (120 criteria) of all exclusion criteria (eTable 3 in Supplement 1). Treatment limitation was an exclusion criterion in 76.0% of studies (57 studies), accounting for 6.7% of all exclusion criteria (97 criteria) (eTable 3 in Supplement 1).

No studies explicitly excluded participants on the basis of sex, gender, sexual orientation, racial or ethnic background, religious background, educational background, socioeconomic status, employment status, or marital status. However, individuals were excluded on the basis of pregnancy (57 studies [76.0%]), lactation (20 studies [26.7%]), fertility or lack of contraceptive use (4 studies [5.3%]), and postmenopausal status (1 study [1.3%]) (eTable 3 in Supplement 1). Individuals were excluded because of communication issues in 18.7% of trials (14 studies), most commonly because of language barriers (eTable 3 in Supplement 1). Participants were excluded because of older age in 8.0% of trials (6 studies) and for cognitive disabilities in 2.7% of trials (2 studies) (eTable 3 in Supplement 1). The majority of exclusion criteria were not explained or justified by the trial authors in the study protocol or the manuscript (979 of 1455 criteria [67.3%]) (eTable 3 in Supplement 1). Our assessment of exclusion criteria as outlined in the Box revealed that most exclusion criteria were strongly (1080 of 1455 criteria [74.2%]) or potentially (297 of 1455 criteria [20.4%]) justifiable.

### Poorly Justified Exclusion Criteria

Overall, 45 of 75 studies (60.0%) had at least 1 poorly justified exclusion criteria, and 5.4% of all exclusion criteria (78 of 1455 criteria) were poorly justified. The most common poorly justified exclusion criteria across trials were pregnancy (19 of 78 criteria [24.4%]), communication barriers (11 of 78 criteria [14.1%]), lactation (10 of 78 criteria [12.8%]), and lack of health insurance (10 of 78 criteria [12.8%]) (eTable 4 in Supplement 1).

### Assessment of Exclusion of Vulnerable Persons in COVID-19 vs Non–COVID-19 Trials

Each trial was assessed on an individual basis regardless of the condition of interest for consistency. Among the 13 COVID-19 trials, 11 (85%) excluded pregnant persons. Exclusion of pregnant persons was strongly justified in 2, potentially justified in 4, and poorly justified in 5 of the 11 trials. For the 62 non–COVID-19 trials, 46 (74%) excluded pregnant persons. Exclusion of pregnant persons in non–COVID-19 trials was strongly justified in 17 of 46 trials, potentially justified in 15 of 46 trials, and poorly justified in 14 of 46 trials. Among the 13 COVID-19 trials, 2 (15%) explicitly excluded incarcerated individuals. For the 62 trials that recruited patients with other conditions, exclusion of incarcerated persons was explicitly stated in 15 (24%). We assessed the exclusion of incarcerated individuals as potentially justifiable across all 17 studies.

## Discussion

In this systematic sampling review of 75 critical care RCTs^12,13,14,15,16,17,18,19,20,21,22,23,24,25,26,27,28,29,30,31,32,33,34,35,36,37,38,39,40,41,42,43,44,45,46,47,48,49,50,51,52,53,54,55,56,57,58,59,60,61,62,63,64,65,66,67,68,69,70,71,72,73,74,75,76,77,78,79,80,81,82,83,84,85,86^ published in high-impact general medicine journals, nearly 95% of the 1455 exclusion criteria were assessed as being strongly or potentially justifiable. Notwithstanding, 60% of trials had at least 1 poorly justified exclusion criterion. The most frequent poorly justified exclusion criteria were pregnant and/or lactating people, those with communication barriers, and individuals lacking health insurance.

The trials included in this review did not explicitly exclude patients on the basis of race or ethnicity, religious beliefs, sex, gender, sexual orientation, employment status, and marital status. However, some trials excluded participants on the basis of sex-related characteristics, most notably those who were pregnant, lactating, and/or fertile without the use of contraceptives. These exclusions are based on historical safety concerns, notably thalidomide-associated birth defects.^87^ Other concerns for including pregnant persons may be due to the length of follow-up required for the parent and fetus, physiological changes during pregnancy that may impact pharmacokinetics and pharmacodynamics, and difficulties obtaining trial insurance for this patient population.^88,89^ It is not surprising that exclusion of pregnant persons was 11% higher in COVID-19 trials compared with non–COVID-19 trials. During the H1N1 pandemic, similar trends were observed because there were limited data on the safety of the H1N1 vaccine in pregnant persons, a high-risk patient population.^90^

Recent US Food and Drug Administration guidelines encourage inclusion of pregnant persons in clinical trials where appropriate.^87^ Although there is a shift toward greater inclusion of pregnant persons, guidelines and legislation vary from region to region, thereby potentially limiting their inclusion. Representation of pregnant persons or those of childbearing potential in RCTs could aid in narrowing the knowledge gap and support health care systems, clinicians, patients, and their families in making evidence-based decisions.

Potential participants were also excluded for several other reasons, including older age, cognitive disabilities, communication barriers, lack of health insurance, and incarceration. Efforts should be made to avoid the broad exclusion of people with disabilities and to make trials accessible for such individuals where feasible and ethical to do so. Similarly, the exclusion of individuals owing to communication barriers is often poorly justified. In this study, patients were excluded from 14 trials because of communication issues. Possible solutions for communication barriers include multilingual research documents, interpretation services, and cultural mediators.^91,92^

In this review, 10 trials denied participation for those who lacked health insurance and 17 trials explicitly excluded incarcerated individuals. Individuals who lack health insurance may represent a population at risk of poor health outcomes and should have opportunities to participate in research trials, with appropriate compensation for study-related expenses.^93,94^ Ethical concerns about coercion and exploitation, as well as logistical challenges with trial follow-up, may result in the exclusion of incarcerated individuals.^95,96^ Incarcerated individuals may be excluded from trials due to local legislation traditionally placed to protect this vulnerable population.^97^ However, broad exclusion may be more harmful, limiting safety data and potential benefit of interventions. During the COVID-19 pandemic, incarcerated people were at higher risk for infection because of overcrowding, poor sanitation, confined spaces, high turnover, and poor access to health care observed in correctional facilities.^98^ Interestingly, our study showed the proportion of COVID-19 trials explicitly excluding incarcerated individuals was 9% lower than non–COVID-19 trials. This is promising as they were a high-risk population for infection with COVID-19. With careful ethical, legal, and logistical considerations, incarcerated persons should be offered participation in research studies where appropriate.

The lack of diversity in research teams may result in narrow perspectives and contribute further to the underrepresentation of diverse patient populations. Women and members of racial and ethnic minority groups, as well as those from LMICs, are underrepresented as research leaders in critical care medicine, highlighting the broader systemic issues of the lack of diversity and inclusion within the field.^10,11,99,100^ In a previous study^99^ of 1398 critical care RCTs published in 12 high-impact journals, only 24.6% of first authors and 16.6% of senior authors were women, and approximately 85% of authors were White. The demographics of first and/or last authors of the studies included in this review corroborate these findings, with only 17.3% of first and/or last authors being female, and the majority being visibly White. This limited diversity may be due to several reasons, including lack of representation of women and other minority groups in critical care medicine, limited access to educational and career opportunities of minoritized individuals in critical care medicine, implicit biases from peer review and journal editors leading to the selection of articles authored by individuals from dominant demographic groups, and lack of diverse mentors in critical care medicine.

We focus on an important aspect of RCT design—namely, eligibility criteria and highlighting the significance of adequate justification for patient selection. The strengths of this study include the collection of comprehensive data from RCTs enrolling diverse critically ill patient populations. In addition, we assessed author demographics and corroborated previous findings, underscoring the need for efforts to improve equity, diversity, and inclusion in critical care medicine, not only for patients but also for professionals in the field. Given the potential subjectivity of the assessment of justification of exclusion criteria, we used established definitions from prior literature to guide our adjudication, which was conducted independently and in duplicate.

### Limitations

Our study has limitations. We only included articles from the top 5 general medical journals by impact factor, because RCTs published in the top journals are most likely to justify their exclusions, given their more-stringent publication requirements.^101,102^ Our search strategy was limited to the MEDLINE database; therefore, relevant publications from additional databases may not have been identified. Our sample size of 75 trials published over 5 years may not be representative of the entire critical care literature. Our findings are consistent with existing research assessing the diversity of authors in critical care medicine publications. However, we acknowledge the limitations in ascribing first and/or last author sex and race, specifically the inability to ascertain author-reported gender and ethnicity that may be fluid, not public, or invisible.

## Conclusions

This study demonstrated that the majority of exclusion criteria in critical care RCTs were strongly justifiable; however, 60% of RCTs had at least 1 poorly justified exclusion criterion. Across poorly justified criteria, the exclusion of pregnant persons was common. The majority of trials had White male first or last authors and few were from LMICs, highlighting the limited global representation across high-impact journals. We encourage investigators to carefully consider exclusion criteria when designing RCTs to improve equitable access to trial participation and the generalizability of trial findings. We also recommend that journals mandate clear justification of all exclusion criteria to aid in broadening participant representation.

## Notes

Supplement 1.
eAppendix.
**eTable 1.** Summary of characteristics for 75 included randomized trials **eTable 2.** Author demographics for 75 randomized trials **eTable 3.** Breakdown of exclusion criteria across all studies **eTable 4.** Summary of poorly justified exclusion criteria
eReferences

Supplement 2.
Canadian Critical Care Trials Group Members

Supplement 3.
Data Sharing Statement

## References

[1] Granholm A, Alhazzani W, Derde LPG, . Randomised clinical trials in critical care: past, present and future. Intensive Care Med. 2022;48(2):164-178. doi:10.1007/s00134-021-06587-9 34853905 PMC8636283

[2] Rothwell PM. External validity of randomised controlled trials: “to whom do the results of this trial apply?”. Lancet. 2005;365(9453):82-93. doi:10.1016/S0140-6736(04)17670-8 15639683

[3] Van Spall HGC, Toren A, Kiss A, Fowler RA. Eligibility criteria of randomized controlled trials published in high-impact general medical journals: a systematic sampling review. JAMA. 2007;297(11):1233-1240. doi:10.1001/jama.297.11.1233 17374817

[4] DeCormier Plosky W, Ne’eman A, Silverman BC, . Excluding people with disabilities from clinical research: eligibility criteria lack clarity and justification. Health Aff (Millwood). 2022;41(10):1423-1432. doi:10.1377/hlthaff.2022.00520 36190895

[5] Krewulak KD, Stelfox HT, Leigh JP, Ely EW, Fiest KM. Incidence and prevalence of delirium subtypes in an adult ICU: a systematic review and meta-analysis. Crit Care Med. 2018;46(12):2029-2035. doi:10.1097/CCM.0000000000003402 30234569

[6] Higgins J, Green S, eds. Cochrane Handbook for Systematic Reviews of Interventions: Cochrane Book Series. Wiley-Blackwell; 2008. doi:10.1002/9780470712184

[7] Orkin AM, Nicoll G, Persaud N, Pinto AD. Reporting of sociodemographic variables in randomized clinical trials, 2014-2020. JAMA Netw Open. 2021;4(6):e2110700. doi:10.1001/jamanetworkopen.2021.10700 34076703 PMC8173372

[8] Granton D, Rodrigues M, Raparelli V, . Sex and gender-based analysis and diversity metric reporting in acute care trials published in high-impact journals: a systematic review. BMJ Open. 2024;14(5):e081118. doi:10.1136/bmjopen-2023-081118 38719297 PMC11103199

[9] World Bank. World Bank country and lending groups: world bank data help desk. Accessed March 28, 2024. https://datahelpdesk.worldbank.org/knowledgebase/articles/906519-world-bank-country-and-lending-groups

[10] Heybati K, Flexman AM, Lorello GR, Mehta S. Outcomes of COVID-19 manuscripts submitted to the *Canadian Journal of Anesthesia*: a retrospective audit of author gender and person of colour status. Can J Anaesth. 2023;70(6):988-994. doi:10.1007/s12630-023-02455-w 37188835 PMC10184964

[11] Mehta S, Ahluwalia N, Heybati K, Burns KEA, Owais S, Cook DJ; Canadian Critical Care Trials Group. Diversity of authors of publications from the Canadian Critical Care Trials Group. Crit Care Med. 2022;50(4):535-542. doi:10.1097/CCM.0000000000005284 34473658

[12] Meyhoff TS, Hjortrup PB, Wetterslev J, ; CLASSIC Trial Group. Restriction of intravenous fluid in ICU patients with septic shock. N Engl J Med. 2022;386(26):2459-2470. doi:10.1056/NEJMoa2202707 35709019

[13] Vourc’h M, Garret C, Gacouin A, ; BACLOREA Study Group. Effect of high-dose baclofen on agitation-related events among patients with unhealthy alcohol use receiving mechanical ventilation: a randomized clinical trial. JAMA. 2021;325(8):732-741. doi:10.1001/jama.2021.0658 33620407 PMC7903253

[14] Sevransky JE, Rothman RE, Hager DN, ; VICTAS Investigators. Effect of vitamin C, thiamine, and hydrocortisone on ventilator- and vasopressor-free days in patients with sepsis: the VICTAS randomized clinical trial. JAMA. 2021;325(8):742-750. doi:10.1001/jama.2020.24505 33620405 PMC7903252

[15] Hughes CG, Mailloux PT, Devlin JW, ; MENDS2 Study Investigators. Dexmedetomidine or propofol for sedation in mechanically ventilated adults with sepsis. N Engl J Med. 2021;384(15):1424-1436. doi:10.1056/NEJMoa2024922 33528922 PMC8162695

[16] Schjørring OL, Klitgaard TL, Perner A, ; HOT-ICU Investigators. Lower or higher oxygenation targets for acute hypoxemic respiratory failure. N Engl J Med. 2021;384(14):1301-1311. doi:10.1056/NEJMoa2032510 33471452

[17] Angus DC, Derde L, Al-Beidh F, ; Writing Committee for the REMAP-CAP Investigators. Effect of hydrocortisone on mortality and organ support in patients with severe COVID-19: the REMAP-CAP COVID-19 corticosteroid domain randomized clinical trial. JAMA. 2020;324(13):1317-1329. doi:10.1001/jama.2020.17022 32876697 PMC7489418

[18] Algera AG, Pisani L, Serpa Neto A, ; Writing Committee and Steering Committee for the RELAx Collaborative Group. Effect of a lower vs higher positive end-expiratory pressure strategy on ventilator-free days in ICU patients without ARDS: a randomized clinical trial. JAMA. 2020;324(24):2509-2520. doi:10.1001/jama.2020.23517 33295981 PMC7726701

[19] Zarbock A, Küllmar M, Kindgen-Milles D, ; RICH Investigators and the Sepnet Trial Group. Effect of regional citrate anticoagulation vs systemic heparin anticoagulation during continuous kidney replacement therapy on dialysis filter life span and mortality among critically ill patients with acute kidney injury: a randomized clinical trial. JAMA. 2020;324(16):1629-1639. doi:10.1001/jama.2020.18618 33095849 PMC7585036

[20] Hernández Martínez G, Rodriguez ML, Vaquero MC, . High-flow oxygen with capping or suctioning for tracheostomy decannulation. N Engl J Med. 2020;383(11):1009-1017. doi:10.1056/NEJMoa2010834 32905673

[21] Tomazini BM, Maia IS, Cavalcanti AB, ; COALITION COVID-19 Brazil III Investigators. Effect of dexamethasone on days alive and ventilator-free in patients with moderate or severe acute respiratory distress syndrome and COVID-19: the CoDEX randomized clinical trial. JAMA. 2020;324(13):1307-1316. doi:10.1001/jama.2020.17021 32876695 PMC7489411

[22] Dequin PF, Heming N, Meziani F, ; CAPE COVID Trial Group and the CRICS-TriGGERSep Network. Effect of hydrocortisone on 21-day mortality or respiratory support among critically ill patients with COVID-19: a randomized clinical trial. JAMA. 2020;324(13):1298-1306. doi:10.1001/jama.2020.16761 32876689 PMC7489432

[23] Bagshaw SM, Wald R, Adhikari NKJ, ; STARRT-AKI Investigators; Canadian Critical Care Trials Group; Australian and New Zealand Intensive Care Society Clinical Trials Group; United Kingdom Critical Care Research Group; Canadian Nephrology Trials Network; Irish Critical Care Trials Group. Timing of initiation of renal-replacement therapy in acute kidney injury. N Engl J Med. 2020;383(3):240-251. doi:10.1056/NEJMoa2000741 32668114

[24] Barrot L, Asfar P, Mauny F, ; LOCO2 Investigators and REVA Research Network. Liberal or conservative oxygen therapy for acute respiratory distress syndrome. N Engl J Med. 2020;382(11):999-1008. doi:10.1056/NEJMoa1916431 32160661

[25] Olsen HT, Nedergaard HK, Strøm T, . Nonsedation or light sedation in critically ill, mechanically ventilated patients. N Engl J Med. 2020;382(12):1103-1111. doi:10.1056/NEJMoa1906759 32068366

[26] Ranieri VM, Pettilä V, Karvonen MK, ; INTEREST Study Group. Effect of intravenous interferon β-1a on death and days free from mechanical ventilation among patients with moderate to severe acute respiratory distress syndrome: a randomized clinical trial. JAMA. 2020;323(8):725-733. doi:10.1001/jama.2019.22525 32065831 PMC12005643

[27] Lamontagne F, Richards-Belle A, Thomas K, . Effect of reduced exposure to vasopressors on 90-day mortality in older critically ill patients with vasodilatory hypotension: a randomized clinical trial. JAMA. 2020;323(10):938-949. doi:10.1001/jama.2020.0930 32049269 PMC7064880

[28] Fujii T, Luethi N, Young PJ, ; VITAMINS Trial Investigators. Effect of vitamin C, hydrocortisone, and thiamine vs hydrocortisone alone on time alive and free of vasopressor support among patients with septic shock: the VITAMINS randomized clinical trial. JAMA. 2020;323(5):423-431. doi:10.1001/jama.2019.22176 31950979 PMC7029761

[29] Mackle D, Bellomo R, Bailey M, ; ICU-ROX Investigators and the Australian and New Zealand Intensive Care Society Clinical Trials Group; ICU-ROX Investigators the Australian and New Zealand Intensive Care Society Clinical Trials Group. Conservative oxygen therapy during mechanical ventilation in the ICU. N Engl J Med. 2020;382(11):989-998. doi:10.1056/NEJMoa1903297 31613432

[30] Subirà C, Hernández G, Vázquez A, . Effect of pressure support vs T-piece ventilation strategies during spontaneous breathing trials on successful extubation among patients receiving mechanical ventilation: a randomized clinical trial. JAMA. 2019;321(22):2175-2182. doi:10.1001/jama.2019.7234 31184740 PMC6563557

[31] Ginde AA, Brower RG, Caterino JM, ; National Heart, Lung, and Blood Institute PETAL Clinical Trials Network. Early high-dose vitamin D_3_ for critically ill, vitamin D-deficient patients. N Engl J Med. 2019;381(26):2529-2540. doi:10.1056/NEJMoa1911124 31826336 PMC7306117

[32] François B, Cariou A, Clere-Jehl R, ; CRICS-TRIGGERSEP Network and the ANTHARTIC Study Group. Prevention of early ventilator-associated pneumonia after cardiac arrest. N Engl J Med. 2019;381(19):1831-1842. doi:10.1056/NEJMoa1812379 31693806

[33] Lascarrou JB, Merdji H, Le Gouge A, ; CRICS-TRIGGERSEP Group. Targeted temperature management for cardiac arrest with nonshockable rhythm. N Engl J Med. 2019;381(24):2327-2337. doi:10.1056/NEJMoa1906661 31577396

[34] Thille AW, Muller G, Gacouin A, ; HIGH-WEAN Study Group and the REVA Research Network. Effect of postextubation high-flow nasal oxygen with noninvasive ventilation vs high-flow nasal oxygen alone on reintubation among patients at high risk of extubation failure: a randomized clinical trial. JAMA. 2019;322(15):1465-1475. doi:10.1001/jama.2019.14901 31577036 PMC6802261

[35] Fowler AA III, Truwit JD, Hite RD, . Effect of vitamin C infusion on organ failure and biomarkers of inflammation and vascular injury in patients with sepsis and severe acute respiratory failure: the CITRIS-ALI randomized clinical trial. JAMA. 2019;322(13):1261-1270. doi:10.1001/jama.2019.11825 31573637 PMC6777268

[36] Garrouste-Orgeas M, Flahault C, Vinatier I, . Effect of an ICU diary on posttraumatic stress disorder symptoms among patients receiving mechanical ventilation: a randomized clinical trial. JAMA. 2019;322(3):229-239. doi:10.1001/jama.2019.9058 31310299 PMC6635906

[37] Shehabi Y, Howe BD, Bellomo R, ; ANZICS Clinical Trials Group and the SPICE III Investigators. Early sedation with dexmedetomidine in critically ill patients. N Engl J Med. 2019;380(26):2506-2517. doi:10.1056/NEJMoa1904710 31112380

[38] Vincent JL, Francois B, Zabolotskikh I, ; SCARLET Trial Group. Effect of a recombinant human soluble thrombomodulin on mortality in patients with sepsis-associated coagulopathy: the SCARLET randomized clinical trial. JAMA. 2019;321(20):1993-2002. doi:10.1001/jama.2019.5358 31104069 PMC6547077

[39] Arabi YM, Al-Hameed F, Burns KEA, ; Saudi Critical Care Trials Group. Adjunctive intermittent pneumatic compression for venous thromboprophylaxis. N Engl J Med. 2019;380(14):1305-1315. doi:10.1056/NEJMoa1816150 30779530

[40] Casey JD, Janz DR, Russell DW, ; PreVent Investigators and the Pragmatic Critical Care Research Group. Bag-mask ventilation during tracheal intubation of critically ill adults. N Engl J Med. 2019;380(9):811-821. doi:10.1056/NEJMoa1812405 30779528 PMC6423976

[41] Hernández G, Ospina-Tascón GA, Damiani LP, ; The ANDROMEDA SHOCK Investigators and the Latin America Intensive Care Network (LIVEN). Effect of a resuscitation strategy targeting peripheral perfusion status vs serum lactate levels on 28-day mortality among patients with septic shock: the ANDROMEDA-SHOCK randomized clinical trial. JAMA. 2019;321(7):654-664. doi:10.1001/jama.2019.0071 30772908 PMC6439620

[42] Cox CE, White DB, Hough CL, . Effects of a personalized web-based decision aid for surrogate decision makers of patients with prolonged mechanical ventilation: a randomized clinical trial. Ann Intern Med. 2019;170(5):285-297. doi:10.7326/M18-2335 30690645 PMC7363113

[43] Laterre PF, Berry SM, Blemings A, ; SEPSIS-ACT Investigators. Effect of selepressin vs placebo on ventilator- and vasopressor-free days in patients with septic shock: the SEPSIS-ACT randomized clinical trial. JAMA. 2019;322(15):1476-1485. doi:10.1001/jama.2019.14607 31577035 PMC6802260

[44] Russell DW, Casey JD, Gibbs KW, ; PREPARE II Investigators and the Pragmatic Critical Care Research Group. Effect of fluid bolus administration on cardiovascular collapse among critically ill patients undergoing tracheal intubation: a randomized clinical trial. JAMA. 2022;328(3):270-279. doi:10.1001/jama.2022.9792 35707974 PMC9204618

[45] Pickkers P, Mehta RL, Murray PT, ; STOP-AKI Investigators. Effect of human recombinant alkaline phosphatase on 7-day creatinine clearance in patients with sepsis-associated acute kidney injury: a randomized clinical trial. JAMA. 2018;320(19):1998-2009. doi:10.1001/jama.2018.14283 30357272 PMC6248164

[46] Azoulay E, Lemiale V, Mokart D, . Effect of high-flow nasal oxygen vs standard oxygen on 28-day mortality in immunocompromised patients with acute respiratory failure: the HIGH randomized clinical trial. JAMA. 2018;320(20):2099-2107. doi:10.1001/jama.2018.14282 30357270 PMC6583581

[47] Simonis FD, Serpa Neto A, Binnekade JM, ; Writing Group for the PReVENT Investigators. Effect of a low vs intermediate tidal volume strategy on ventilator-free days in intensive care unit patients without ARDS: a randomized clinical trial. JAMA. 2018;320(18):1872-1880. doi:10.1001/jama.2018.14280 30357256 PMC6248136

[48] Krag M, Marker S, Perner A, ; SUP-ICU trial group. Pantoprazole in patients at risk for gastrointestinal bleeding in the ICU. N Engl J Med. 2018;379(23):2199-2208. doi:10.1056/NEJMoa1714919 30354950

[49] Perkins GD, Mistry D, Gates S, ; Breathe Collaborators. Effect of protocolized weaning with early extubation to noninvasive ventilation vs invasive weaning on time to liberation from mechanical ventilation among patients with respiratory failure: the breathe randomized clinical trial. JAMA. 2018;320(18):1881-1888. doi:10.1001/jama.2018.13763 30347090 PMC6248131

[50] Heyland DK, Patel J, Compher C, ; EFFORT Protein Trial Team. The effect of higher protein dosing in critically ill patients with high nutritional risk (EFFORT Protein): an international, multicentre, pragmatic, registry-based randomised trial. Lancet. 2023;401(10376):568-576. doi:10.1016/S0140-6736(22)02469-2 36708732

[51] Girard TD, Exline MC, Carson SS, ; MIND-USA Investigators. Haloperidol and ziprasidone for treatment of delirium in critical illness. N Engl J Med. 2018;379(26):2506-2516. doi:10.1056/NEJMoa1808217 30346242 PMC6364999

[52] Chapman M, Peake SL, Bellomo R, ; TARGET Investigators, for the ANZICS Clinical Trials Group. Energy-dense versus routine enteral nutrition in the critically ill. N Engl J Med. 2018;379(19):1823-1834. doi:10.1056/NEJMoa1811687 30346225

[53] Barbar SD, Clere-Jehl R, Bourredjem A, ; IDEAL-ICU Trial Investigators and the CRICS TRIGGERSEP Network. Timing of renal-replacement therapy in patients with acute kidney injury and sepsis. N Engl J Med. 2018;379(15):1431-1442. doi:10.1056/NEJMoa1803213 30304656

[54] Dellinger RP, Bagshaw SM, Antonelli M, ; EUPHRATES Trial Investigators. Effect of targeted polymyxin B hemoperfusion on 28-day mortality in patients with septic shock and elevated endotoxin level: the EUPHRATES randomized clinical trial. JAMA. 2018;320(14):1455-1463. doi:10.1001/jama.2018.14618 30304428 PMC6233793

[55] Fossat G, Baudin F, Courtes L, . Effect of in-bed leg cycling and electrical stimulation of the quadriceps on global muscle strength in critically ill adults: a randomized clinical trial. JAMA. 2018;320(4):368-378. doi:10.1001/jama.2018.9592 30043066 PMC6583091

[56] Jaber S, Paugam C, Futier E, ; BICAR-ICU Study Group. Sodium bicarbonate therapy for patients with severe metabolic acidaemia in the intensive care unit (BICAR-ICU): a multicentre, open-label, randomised controlled, phase 3 trial. Lancet. 2018;392(10141):31-40. doi:10.1016/S0140-6736(18)31080-8 29910040

[57] Annane D, Renault A, Brun-Buisson C, ; CRICS-TRIGGERSEP Network. Hydrocortisone plus fludrocortisone for adults with septic shock. N Engl J Med. 2018;378(9):809-818. doi:10.1056/NEJMoa1705716 29490185

[58] van Meenen DMP, van der Hoeven SM, Binnekade JM, . Effect of on-demand vs routine nebulization of acetylcysteine with salbutamol on ventilator-free days in intensive care unit patients receiving invasive ventilation: a randomized clinical trial. JAMA. 2018;319(10):993-1001. doi:10.1001/jama.2018.0949 29486489 PMC5885882

[59] van den Boogaard M, Slooter AJC, Brüggemann RJM, ; REDUCE Study Investigators. Effect of haloperidol on survival among critically ill adults with a high risk of delirium: the REDUCE randomized clinical trial. JAMA. 2018;319(7):680-690. doi:10.1001/jama.2018.0160 29466591 PMC5839284

[60] Venkatesh B, Finfer S, Cohen J, ; ADRENAL Trial Investigators and the Australian–New Zealand Intensive Care Society Clinical Trials Group. Adjunctive glucocorticoid therapy in patients with septic shock. N Engl J Med. 2018;378(9):797-808. doi:10.1056/NEJMoa1705835 29347874

[61] Reignier J, Boisramé-Helms J, Brisard L, ; NUTRIREA-2 Trial Investigators; Clinical Research in Intensive Care and Sepsis (CRICS) group. Enteral versus parenteral early nutrition in ventilated adults with shock: a randomised, controlled, multicentre, open-label, parallel-group study (NUTRIREA-2). Lancet. 2018;391(10116):133-143. doi:10.1016/S0140-6736(17)32146-3 29128300

[62] Frat JP, Quenot JP, Badie J, ; SOHO-COVID Study Group and the REVA Network. Effect of high-flow nasal cannula oxygen vs standard oxygen therapy on mortality in patients with respiratory failure due to COVID-19: the SOHO-COVID randomized clinical trial. JAMA. 2022;328(12):1212-1222. doi:10.1001/jama.2022.15613 36166027 PMC9516287

[63] Heyland DK, Wibbenmeyer L, Pollack J, ; RE-ENERGIZE Trial Team. A randomized trial of enteral glutamine for treatment of burn injuries. N Engl J Med. 2022;387(11):1001-1010. doi:10.1056/NEJMoa2203364 36082909

[64] Lamontagne F, Masse MH, Menard J, ; LOVIT Investigators and the Canadian Critical Care Trials Group. Intravenous vitamin C in adults with sepsis in the intensive care unit. N Engl J Med. 2022;386(25):2387-2398. doi:10.1056/NEJMoa2200644 35704292

[65] Alhazzani W, Parhar KKS, Weatherald J, ; COVI-PRONE Trial Investigators and the Saudi Critical Care Trials Group. Effect of awake prone positioning on endotracheal intubation in patients with COVID-19 and acute respiratory failure: a randomized clinical trial. JAMA. 2022;327(21):2104-2113. doi:10.1001/jama.2022.7993 35569448 PMC9108999

[66] Finfer S, Micallef S, Hammond N, ; PLUS Study Investigators and the Australian New Zealand Intensive Care Society Clinical Trials Group. Balanced multielectrolyte solution versus saline in critically ill adults. N Engl J Med. 2022;386(9):815-826. doi:10.1056/NEJMoa2114464 35041780

[67] Bradbury CAJ, Lawler PR, Stanworth SJ, ; REMAP-CAP Writing Committee for the REMAP-CAP Investigators. Effect of antiplatelet therapy on survival and organ support-free days in critically ill patients with COVID-19: a randomized clinical trial. JAMA. 2022;327(13):1247-1259. doi:10.1001/jama.2022.2910 35315874 PMC8941448

[68] Ruijter BJ, Keijzer HM, Tjepkema-Cloostermans MC, ; TELSTAR Investigators. Treating rhythmic and periodic EEG patterns in comatose survivors of cardiac arrest. N Engl J Med. 2022;386(8):724-734. doi:10.1056/NEJMoa2115998 35196426

[69] Levy B, Girerd N, Amour J, ; HYPO-ECMO Trial Group and the International ECMO Network (ECMONet). Effect of moderate hypothermia vs normothermia on 30-day mortality in patients with cardiogenic shock receiving venoarterial extracorporeal membrane oxygenation: a randomized clinical trial. JAMA. 2022;327(5):442-453. doi:10.1001/jama.2021.24776 35103766 PMC8808325

[70] INSPIRATION-S Investigators. Atorvastatin versus placebo in patients with covid-19 in intensive care: randomized controlled trial. BMJ. 2022;376:e068407. doi:10.1136/bmj-2021-068407 34996756 PMC11785411

[71] Zampieri FG, Machado FR, Biondi RS, ; BaSICS Investigators and the BRICNet Members. Effect of intravenous fluid treatment with a balanced solution vs 0.9% saline solution on mortality in critically ill patients: the BaSICS randomized clinical trial. JAMA. 2021;326(9):1-12. doi:10.1001/jama.2021.11684 34375394 PMC8356144

[72] McNamee JJ, Gillies MA, Barrett NA, ; REST Investigators. Effect of lower tidal volume ventilation facilitated by extracorporeal carbon dioxide removal vs standard care ventilation on 90-day mortality in patients with acute hypoxemic respiratory failure: the REST randomized clinical trial. JAMA. 2021;326(11):1013-1023. doi:10.1001/jama.2021.13374 34463700 PMC8408762

[73] Goligher EC, Bradbury CA, McVerry BJ, ; REMAP-CAP Investigators; ACTIV-4a Investigators; ATTACC Investigators. Therapeutic anticoagulation with heparin in critically ill patients with Covid-19. N Engl J Med. 2021;385(9):777-789. doi:10.1056/NEJMoa2103417 34351722 PMC8362592

[74] Driver BE, Semler MW, Self WH, ; BOUGIE Investigators and the Pragmatic Critical Care Research Group. Effect of use of a bougie vs endotracheal tube with stylet on successful intubation on the first attempt among critically ill patients undergoing tracheal intubation: a randomized clinical trial. JAMA. 2021;326(24):2488-2497. doi:10.1001/jama.2021.22002 34879143 PMC8655668

[75] Ospina-Tascón GA, Calderón-Tapia LE, García AF, ; HiFLo-Covid Investigators. Effect of high-flow oxygen therapy vs conventional oxygen therapy on invasive mechanical ventilation and clinical recovery in patients with severe COVID-19: a randomized clinical trial. JAMA. 2021;326(21):2161-2171. doi:10.1001/jama.2021.20714 34874419 PMC8652598

[76] Andersen-Ranberg NC, Poulsen LM, Perner A, ; AID-ICU Trial Group. Haloperidol for the treatment of delirium in ICU patients. N Engl J Med. 2022;387(26):2425-2435. doi:10.1056/NEJMoa2211868 36286254

[77] Estcourt LJ, Turgeon AF, McQuilten ZK, ; Writing Committee for the REMAP-CAP Investigators. Effect of convalescent plasma on organ support-free days in critically ill patients with COVID-19: a randomized clinical trial. JAMA. 2021;326(17):1690-1702. doi:10.1001/jama.2021.18178 34606578 PMC8491132

[78] Zampieri FG, Machado FR, Biondi RS, ; BaSICS investigators and the BRICNet members. Effect of slower vs faster intravenous fluid bolus rates on mortality in critically ill patients: the BaSICS randomized clinical trial. JAMA. 2021;326(9):830-838. doi:10.1001/jama.2021.11444 34547081 PMC8356145

[79] Johnstone J, Meade M, Lauzier F, ; Prevention of Severe Pneumonia and Endotracheal Colonization Trial (PROSPECT) Investigators and the Canadian Critical Care Trials Group. Effect of probiotics on incident ventilator-associated pneumonia in critically ill patients: a randomized clinical trial. JAMA. 2021;326(11):1024-1033. doi:10.1001/jama.2021.13355 34546300 PMC8456390

[80] Gelissen H, de Grooth HJ, Smulders Y, . Effect of low-normal vs high-normal oxygenation targets on organ dysfunction in critically ill patients: a randomized clinical trial. JAMA. 2021;326(10):940-948. doi:10.1001/jama.2021.13011 34463696 PMC8408761

[81] Hodgson CL, Bailey M, Bellomo R, ; TEAM Study Investigators and the ANZICS Clinical Trials Group. Early active mobilization during mechanical ventilation in the ICU. N Engl J Med. 2022;387(19):1747-1758. doi:10.1056/NEJMoa2209083 36286256

[82] Roquilly A, Moyer JD, Huet O, ; Atlanrea Study Group and the Société Française d’Anesthésie Réanimation (SFAR) Research Network. Effect of continuous infusion of hypertonic saline vs standard care on 6-month neurological outcomes in patients with traumatic brain injury: the COBI randomized clinical trial. JAMA. 2021;325(20):2056-2066. doi:10.1001/jama.2021.5561 34032829 PMC8150692

[83] Gaudry S, Hajage D, Martin-Lefevre L, . Comparison of two delayed strategies for renal replacement therapy initiation for severe acute kidney injury (AKIKI 2): a multicentre, open-label, randomised, controlled trial. Lancet. 2021;397(10281):1293-1300. doi:10.1016/S0140-6736(21)00350-0 33812488

[84] Gordon AC, Mouncey PR, Al-Beidh F, ; REMAP-CAP Investigators. Interleukin-6 receptor antagonists in critically ill patients with Covid-19. N Engl J Med. 2021;384(16):1491-1502. doi:10.1056/NEJMoa2100433 33631065 PMC7953461

[85] Grieco DL, Menga LS, Cesarano M, ; COVID-ICU Gemelli Study Group. Effect of helmet noninvasive ventilation vs high-flow nasal oxygen on days free of respiratory support in patients with COVID-19 and moderate to severe hypoxemic respiratory failure: the HENIVOT randomized clinical trial. JAMA. 2021;325(17):1731-1743. doi:10.1001/jama.2021.4682 33764378 PMC7995134

[86] Sadeghipour P, Talasaz AH, Rashidi F, ; INSPIRATION Investigators. Effect of intermediate-dose vs standard-dose prophylactic anticoagulation on thrombotic events, extracorporeal membrane oxygenation treatment, or mortality among patients with COVID-19 admitted to the intensive care unit: the INSPIRATION randomized clinical trial. JAMA. 2021;325(16):1620-1630. doi:10.1001/jama.2021.4152 33734299 PMC7974835

[87] US Food and Drug Administration, Center for Drug Evaluation Research. Division of Pediatric and Maternal Health—clinical trials in pregnant women. December 20, 2019. Accessed March 27, 2024. https://www.fda.gov/drugs/development-resources/division-pediatric-and-maternal-health-clinical-trials-pregnant-women

[88] Blehar MC, Spong C, Grady C, Goldkind SF, Sahin L, Clayton JA. Enrolling pregnant women: issues in clinical research. Womens Health Issues. 2013;23(1):e39-e45. doi:10.1016/j.whi.2012.10.003 23312713 PMC3547525

[89] National Academies of Sciences, Engineering, and Medicine; Health and Medicine Division; Board on Health Sciences Policy; Forum on Drug Discovery, Development, and Translation. Shore C, March A, Wizemann T. The Legal Landscape. In: Inclusion of Pregnant and Lactating Persons in Clinical Trials: Proceedings of a Workshop. National Academies Press; 2022. Accessed November 9, 2024. https://www.ncbi.nlm.nih.gov/books/NBK589758/36520983

[90] Goldkind SF, Sahin L, Gallauresi B. Enrolling pregnant women in research: lessons from the H1N1 influenza pandemic. N Engl J Med. 2010;362(24):2241-2243. doi:10.1056/NEJMp1003462 20554981

[91] O’Hearn K, Gibson J, Krewulak K, ; Canadian Critical Care Trials Group. Consent models in Canadian critical care randomized controlled trials: a scoping review. Can J Anaesth. 2022;69(4):513-526. doi:10.1007/s12630-021-02176-y 34907503

[92] Russell AM, Shepherd V, Woolfall K, . Complex and alternate consent pathways in clinical trials: methodological and ethical challenges encountered by underserved groups and a call to action. Trials. 2023;24(1):151. doi:10.1186/s13063-023-07159-6 36855178 PMC9973248

[93] Cho HL, Danis M, Grady C. The ethics of uninsured participants accessing healthcare in biomedical research: a literature review. Clin Trials. 2018;15(5):509-521. doi:10.1177/1740774518792277 30070143 PMC6133717

[94] Lyon SM, Benson NM, Cooke CR, Iwashyna TJ, Ratcliffe SJ, Kahn JM. The effect of insurance status on mortality and procedural use in critically ill patients. Am J Respir Crit Care Med. 2011;184(7):809-815. doi:10.1164/rccm.201101-0089OC 21700910 PMC3208649

[95] Coughlin SS, Lewis SR, Smith SA. Ethical and social issues in health research involving incarcerated people. J Health Care Poor Underserved. 2016;27(2A):18-28. doi:10.1353/hpu.2016.0053 27133509 PMC4862602

[96] Khatri UG, Winkelman TNA. Strengthening the Medicaid Reentry Act: supporting the health of people who are incarcerated. N Engl J Med. 2022;386(16):1488-1490. doi:10.1056/NEJMp2119571 35081297

[97] Welch MJ, Lally R, Miller JE, . The ethics and regulatory landscape of including vulnerable populations in pragmatic clinical trials. Clin Trials. 2015;12(5):503-510. doi:10.1177/1740774515597701 26374681 PMC4662375

[98] Strassle C, Jardas E, Ochoa J, . Covid-19 vaccine trials and incarcerated people: the ethics of inclusion. N Engl J Med. 2020;383(20):1897-1899. doi:10.1056/NEJMp2025955 33085884

[99] Chander S, Luhana S, Sadarat F, Leys L, Parkash O, Kumari R. Gender and racial differences in first and senior authorship of high-impact critical care randomized controlled trial studies from 2000 to 2022. Ann Intensive Care. 2023;13(1):56. doi:10.1186/s13613-023-01157-2 37368060 PMC10299980

[100] Eldridge L, Garton EM, Duncan K, Gopal S. Authorship of publications supported by NCI-funded grants involving low- and middle-income countries. JAMA Netw Open. 2024;7(3):e243215. doi:10.1001/jamanetworkopen.2024.3215 38551565 PMC10980966

[101] Rubin E; Editors. Striving for diversity in research studies. N Engl J Med. 2021;385(15):1429-1430. doi:10.1056/NEJMe2114651 34516052

[102] Schwartz AL, Alsan M, Morris AA, Halpern SD. Why diverse clinical trial participation matters. N Engl J Med. 2023;388(14):1252-1254. doi:10.1056/NEJMp2215609 37017480

