# Supplementary material for: Eligibility Criteria of Randomized Clinical Trials in Critical Care Medicine
Source: JAMA Netw Open. 2025 Jan 17;8(1):e2454944. doi: 10.1001/jamanetworkopen.2024.54944 (PMC11742542; doi:10.1001/jamanetworkopen.2024.54944)
Supplement: Supplement 1. — eAppendix. eTable 1. Summary of characteristics for 75 included randomized trials eTable 2. Author demographics for 75 randomized trials eTable 3. Breakdown of exclusion criteria across all studies eTable 4. Summary of poorly justified exclusion criteria eReferences [file jamanetwopen-e2454944-s001.pdf]

Supplemental Online Content

Heirali A, Heybati K, Sereeyotin J, et al; Canadian Critical Care Trials Group. Eligibility criteria of randomized clinical trials in critical care medicine. *JAMA Netw Open*. 2025;8(1):e245944. doi:10.1001/jamanetworkopen.2024.54944

eAppendix
eTable 1. Summary of characteristics for 75 included randomized trials
eTable 2. Author demographics for 75 randomized trials
eTable 3. Breakdown of exclusion criteria across all studies
eTable 4. Summary of poorly justified exclusion criteria
eReferences

This supplemental material has been provided by the authors to give readers additional information about their work.

© 2025 Heirali A et al. *JAMA Network Open.*

eAppendix.

A total of 225 studies were identified using our search strategy, 75 of which met eligibility criteria (Figure). Most of the studies were published in JAMA (41 studies [54.7%][1–41] and NEJM (28 studies [37.3%][42–69]) (eTable 1 in Supplement 1). The majority of trials were multicentre (74 studies, 98.7%),[1-23,25–75] and multi-national including participants from three or more countries (26 studies, 34.7%)[3,8,10,15–17,19,28,29,35,37,42,44,46,48,53,54,56,61–63,66–69,71] (Table S1). Patients were enrolled from 46 unique countries; the majority of which were high-income countries (32 of 46 countries, 69.6%), and only one study (2.2%)[29] enrolled patients from a low-income country (Table S1). Most of the studies were phase 3 (29 studies, 38.7%)[1–3,6–8,15,29,31,32,35,36,39,41,43,47,50,51,53,57–60,62,63,68,69,72,74], open-label (41 studies, 54.7%),[3–6,9–12,14,16,18,20–22,25,28–30,32–36,38–41,45–48,53–55,59,65,66,69,72,73,75] drug trials (33 studies, 44.0%),[1–3,5–8,10,13,15,17,19,23,26,29,31,37,39,43,50,51,53,56,57,60–64,67,69,72,74] and compared interventions to standard of care (42 studies, 56.0%)[3,4,6,9,11,12,14,20–25,27,28,30,32,34,36,39,41–46,49,52–55,58,64–66,68–73,75] (Table S1). The majority of trials were publicly funded (59 studies, 78.7%)[1,3–5,7,9–12,14,16,18,20,22,24,25,27–41,43–51,54–68,70,72–75] (Table S1). The most common conditions of interest were respiratory (21 studies, 28.0%),[4,11,12,14,18,20–22,25,32,33,37,44,45,48,49,51,53,55,68,70] followed by sepsis (14 studies, 18.7%)[2,10,13,15–17,19,23,42,43,59–61,63] (Table S1).

© 2025 Heirali A et al. *JAMA Network Open*.

eTable 1: Summary of characteristics for 75 included randomized trials

Variables	Number of Studies N (%)
**Journal**	
**JAMA**	41 (54.7)
**NEJM**	28 (37.3)
**Lancet**	4 (5.3)
**Annals of Internal Medicine**	1 (1.3)
**BMJ**	1 (1.3)
**Publication year**	
**2018**	16 (21.3)
**2019**	14 (18.7)
**2020**	13 (17.3)
**2021**	18 (24.0)
**2022**	13 (17.3)
**2023**	1 (1.3)
**Funding**	
**Public**	59 (78.7)
**Mixed**	8 (10.7)
**Private**	6 (8.0)
**None**	1 (1.3)
**Unclear**	1 (1.3)
**Number of sites**	
**Multi-center**	74 (98.7)
**Single centre**	1 (1.3)
**Number of sites per study, median (IQR), range**	29.5 (13.0-48.3), 7-43
**Country/countries of enrollment**	
**Multinational (enrolment in ≥3 countries)**	26 (34.7)
**France**	17 (22.7)
**United States**	9 (12.0)
**The Netherlands**	5 (6.7)
**Australia and New Zealand**	3 (4.0)
**Brazil**	3 (4.0)
**United Kingdom**	3 (4.0)
**Iran**	2 (2.7)
**Spain**	2 (2.7)
**Canada and United States**	1 (1.3)
**Colombia**	1 (1.3)
**Germany**	1 (1.3)
**Italy**	1 (1.3)
**The Netherlands and Belgium**	1 (1.3)
**Enrollment from low, middle, high income country†2**	
**High-income**	32 (69.6)
**Middle-income**	13 (28.2)
**Low-income**	1 (2.2)
**Study Phase**	
**II**	3 (4.0)
**2b/3**	1 (1.3)
**III**	29 (38.7)
**IV**	11 (14.7)
**NA**	28 (37.3)

© 2025 Heirali A et al. *JAMA Network Open*.

**Other**	1 (1.3)
**Unspecified**	2 (2.7)
**Comparator**	
**Standard of Care**	42 (56.0)
**Placebo**	21 (28.0)
**Other intervention**	10 (13.3)
**No intervention control**	2 (2.7)
**Blinding**	
**None**	41 (54.7)
**Single**	11 (14.7)
**Double**	23 (30.7)
**Condition of interest***	
**Respiratory**	21 (28.0)
**Sepsis**	14 (18.7)
**COVID-19**	13 (17.3)
**Acute Kidney Injury**	3 (4.0)
**Delirium**	3 (4.0)
**Broad critical illness**	3 (4.0)
**Acute Respiratory Distress Syndrome**	2 (2.7)
**Nutrition**	2 (2.7)
**Neurological**	2 (2.7)
**Gastro-intestinal**	2 (2.7)
**Psychological**	1 (1.3)
**Musculoskeletal**	1 (1.3)
**Hematological**	1 (1.3)
**Other**	7 (9.3)
**Number of Participants**	
**≤250**	8 (10.7%)
**251 to 500**	20 (26.7%)
**501 to 1000**	22 (29.3%)
**1001 to 3000**	17 (22.7%)
**≥3001**	8 (10.7%)
**Median (quartile 1, quartile 3)**	710 (387, 1285)
**Mean (SD, minimum, maximum)**	1338.4 (1930.6, 110, 11052)
**Type of Intervention**	
**Drug**	33 (44.0)
**Protocol**	22 (29.3)
**Ventilation Strategy**	13 (17.3)
**Device**	2 (2.7)
**Drug and Protocol**	1 (1.3)
**Sedation Strategy**	1 (1.3)
**Other (e.g., Web based decision aid, ICU diary, convalescent plasma)**	3 (4.0)

Abbreviations: BMJ =British Medical Journal; JAMA =Journal of the American Medical Association; NEJM = New England Journal of Medicine; Private = funding provided by industry; *Condition of interest=condition required for enrollment in trial; ICU=Intensive care unit; Country of enrollment includes all countries where patients were recruited from. Most studies enrolled patients from three or more countries, while some studies recruited patients solely from one country and a few studies recruited patients from two countries; No intervention controls =

© 2025 Heirali A et al. *JAMA Network Open*.

study that compared intervention group to patients that did not receive intervention, standard of care, or placebo.; †: N= 46 unique countries across all included trials and 5 did not specify the countries of enrolment.

[2] World Bank Country and Lending Groups – World Bank Data Help Desk. Available at: https://datahelpdesk.worldbank.org/knowledgebase/articles/906519-world-bank-country-and-lending-groups. (Accessed: 8th December 2023)

© 2025 Heirali A et al. *JAMA Network Open*.

eTable 2: Author demographics for 75 randomized trials

Variable	First author N (%)	Last author N (%)
**Sex***		
**Female**	13 (17.3)	13 (17.3)
**Male**	61 (81.3)	62 (82.7)
**Female and male co-first authors**	1 (1.3)	NA
**Race**		
**POC**	15 (20.0)	13 (17.3)
**White**	60 (80.0)	62 (82.7)
**Country**		
**Australia**	5 (6.7)	4 (5.3)
**Belgium**	2 (2.7)	0 (0.0)
**Brazil**	3 (4.0)	4 (5.3)
**Canada**	8 (10.7)	5 (6.7)
**Chile**	1 (1.3)	1 (1.3)
**Colombia**	1 (1.3)	0 (0)
**Denmark**	5 (6.7)	5 (6.7)
**France**	17 (22.7)	17 (22.7)
**Germany**	1 (1.3)	2 (2.7)
**Iran**	1 (1.3)	2 (2.7)
**Italy**	2 (2.7)	1 (1.3)
**Japan**	1 (1.3)	0 (0.0)
**New Zealand**	1 (1.3)	3 (4.0)
**Saudi Arabia**	1 (1.3)	2 (2.7)
**Spain**	2 (2.7)	2 (2.7)
**The Netherlands**	7 (9.3)	8 (10.7)
**United Kingdom**	6 (8.0)	7 (9.3)
**United States**	11 (14.7)	12 (16.0)

Abbreviations: * Six publications share three first authors, and 21 publications share nine last authors, indicating multiple contributions by the same authors. POC = Person of color, defined as people who are not Aboriginal/Indigenous, who are non-Caucasian in race or non-white in color according to the Canadian Employment Equity Act[3], available at: https://laws-lois.justice.gc.ca/eng/acts/e-5.401/. (Accessed: 25th October 2023)

© 2025 Heirali A et al. *JAMA Network Open*.

eTable 3: Breakdown of exclusion criteria across all studies

Type of Exclusion Criteria	Total Number of Studies N=75	Total Number of Exclusion Criteria N=1455
**Breastfeeding**	20 (26.7)	20 (1.4)
**Co-intervention present that may confound treatment effect**	31 (41.3)	52 (3.6)
**Cognitive impairment**	2 (2.7)	2 (0.1)
**Communication barrier**	14 (18.7)	25 (1.7)
**Eligible but not enrolled/randomized/followed**	6 (8.0)	6 (0.4)
**Family dynamic issues**	2 (2.7)	2 (0.1)
**Fertile or lack contraception**	4 (5.3)	4 (0.3)
**Hematologic comorbidity**	1 (1.3)	1 (0.1)
**Hepatic comorbidity**	1 (1.3)	1 (0.1)
**Incarceration**	17 (22.7)	17 (1.2)
**Independent condition that may confound treatment effect**	30 (40.0)	72 (4.9)
**Ineligible before randomization**	1 (1.3)	1 (0.1)
**Infectious disease comorbidity**	1 (1.3)	1 (0.1)
**Lack of health insurance**	10 (13.3)	10 (0.7)
**Logistic reasons**	27 (36.0)	27 (1.9)
**May not adhere**	1 (1.3)	2 (0.1)
**May not complete follow up**	4 (5.3)	5 (0.3)
**Medication related**	4 (5.3)	5 (0.3)
**Missed**	6 (8.0)	6 (0.4)
**Missed due to clinical acuity**	2 (2.7)	2 (0.1)
**Neurologic comorbidity**	1 (1.3)	1 (0.1)
**No endotoxin assay activity/endotoxin activity <0.6**	2 (2.7)	2 (0.1)
**Not at risk for outcome**	18 (24.0)	24 (1.6)
**Not enrolled for unknown reasons/unknown**	3 (4.0)	3 (0.2)
**Not likely to have the condition of interest**	32 (42.7)	71 (4.9)
**Old age**	6 (8.0)	6 (0.4)
**Omitted by investigators**	3 (4.0)	3 (0.2)
**Opinion of physician**	30 (40.0)	32 (2.2)
**Other reasons**	6 (8.0)	6 (0.4)
**Participation in another trial**	43 (57.3)	44 (3.0)
**Persons with any kind of dependency on the investigator or employed by the sponsor or investigator**	2 (2.7)	2 (0.1)
**Planned operator is a clinician expected to routinely perform tracheal intubation in the participating unit**	3 (4.0)	3 (0.2)
**Pregnancy**	57 (76.0)	57 (3.9)
**Prior participation in current trial**	29 (38.7)	29 (2.0)
**Received the intervention in the past**	21 (28.0)	26 (1.8)
**Receiving full time residential nursing care**	1 (1.3)	1 (0.1)
**Recent burns**	6 (8.0)	6 (0.4)
**Recent surgery**	4 (5.3)	10 (0.7)
**Recent trauma**	1 (1.3)	1 (0.1)
**Refusal to participate**	45 (60.0)	69 (4.7)
**Short life expectancy**	41 (54.7)	46 (3.2)
**Substance misuse**	1 (1.3)	1 (0.1)

© 2025 Heirali A et al. *JAMA Network Open*.

**This is not the first ICU admission in the index hospital admission**	1 (1.3)	1 (0.1)
**Timing**	52 (69.3)	83 (5.7)
**Transferred/expected to be transferred**	7 (9.3)	7 (0.5)
**Treatment limit decision**	57 (76.0)	97 (6.7)
**Type of disease that is unlikely to respond to treatment**	23 (30.7)	48 (3.3)
**Unable to grant informed consent**	64 (85.3)	120 (8.2)
**Unacceptable risk of adverse reaction to the intervention**	71 (94.7)	302 (20.8)
**Unacceptable risk of withholding intervention**	36 (48.0)	85 (5.8)
**Under or overweight**	8 (10.7)	8 (0.5)

Data presented as N (%)

© 2025 Heirali A et al. *JAMA Network Open*.

eTable 4: Summary of poorly justified exclusion criteria

Type of Exclusion Criteria	Total Number N=78
**Pregnancy**	19 (24.4)
**Communication barrier**	11 (14.1)
**Breastfeeding**	10 (12.8)
**Lack of health insurance**	10 (12.8)
**Missed**	6 (7.7)
**Eligible but not enrolled/followed/randomized**	4 (5.1)
**Old age**	4 (5.1)
**Unacceptable risk of withholding the intervention**	3 (3.8)
**Fertile or lack contraception**	2 (2.6)
**Timing**	2 (2.6)
**Not enrolled for unknown reasons/unknown**	2 (2.6)
**Previously received the intervention**	1 (1.3)
**Under or overweight**	1 (1.3)
**Ineligible before randomization**	1 (1.3)
**Omitted by investigators**	1 (1.3)
**Receiving full time residential nursing care**	1 (1.3)

Data presented as N (%)

© 2025 Heirali A et al. *JAMA Network Open*.

eREFERENCES

1. Vourc'h M, Garret C, Gacouin A, et al. Effect of High-Dose Baclofen on Agitation-Related Events Among Patients With Unhealthy Alcohol Use Receiving Mechanical Ventilation: A Randomized Clinical Trial. *JAMA*. 2021;325(8):732-741. doi:10.1001/jama.2021.0658

2. Sevransky JE, Rothman RE, Hager DN, et al. Effect of Vitamin C, Thiamine, and Hydrocortisone on Ventilator- and Vasopressor-Free Days in Patients With Sepsis: The VICTAS Randomized Clinical Trial. *JAMA*. 2021;325(8):742-750. doi:10.1001/jama.2020.24505

3. Angus DC, Derde L, Al-Beidh F, et al. Effect of Hydrocortisone on Mortality and Organ Support in Patients With Severe COVID-19: The REMAP-CAP COVID-19 Corticosteroid Domain Randomized Clinical Trial. *JAMA*. 2020;324(13):1317-1329. doi:10.1001/jama.2020.17022

4. Writing Committee and Steering Committee for the RELAx Collaborative Group, Algera AG, Pisani L, et al. Effect of a Lower vs Higher Positive End-Expiratory Pressure Strategy on Ventilator-Free Days in ICU Patients Without ARDS: A Randomized Clinical Trial. *JAMA*. 2020;324(24):2509-2520. doi:10.1001/jama.2020.23517

5. Zarbock A, Küllmar M, Kindgen-Milles D, et al. Effect of Regional Citrate Anticoagulation vs Systemic Heparin Anticoagulation During Continuous Kidney Replacement Therapy on Dialysis Filter Life Span and Mortality Among Critically Ill Patients With Acute Kidney Injury: A Randomized Clinical Trial. *JAMA*. 2020;324(16):1629-1639. doi:10.1001/jama.2020.18618

6. Tomazini BM, Maia IS, Cavalcanti AB, et al. Effect of Dexamethasone on Days Alive and Ventilator-Free in Patients With Moderate or Severe Acute Respiratory Distress Syndrome and COVID-19: The CoDEX Randomized Clinical Trial. *JAMA*. 2020;324(13):1307-1316. doi:10.1001/jama.2020.17021

7. Dequin PF, Heming N, Meziani F, et al. Effect of Hydrocortisone on 21-Day Mortality or Respiratory Support Among Critically Ill Patients With COVID-19: A Randomized Clinical Trial. *JAMA*. 2020;324(13):1298-1306. doi:10.1001/jama.2020.16761

8. Ranieri VM, Pettilä V, Karvonen MK, et al. Effect of Intravenous Interferon β-1a on Death and Days Free From Mechanical Ventilation Among Patients With Moderate to Severe Acute Respiratory Distress Syndrome: A Randomized Clinical Trial. *JAMA*. 2020;323(8):725-733. doi:10.1001/jama.2019.22525

9. Lamontagne F, Richards-Belle A, Thomas K, et al. Effect of Reduced Exposure to Vasopressors on 90-Day Mortality in Older Critically Ill Patients With Vasodilatory Hypotension: A Randomized Clinical Trial. *JAMA*. 2020;323(10):938-949. doi:10.1001/jama.2020.0930

10. Fujii T, Luethi N, Young PJ, et al. Effect of Vitamin C, Hydrocortisone, and Thiamine vs Hydrocortisone Alone on Time Alive and Free of Vasopressor Support Among Patients With Septic Shock: The VITAMINS Randomized Clinical Trial. *JAMA*. 2020;323(5):423-431. doi:10.1001/jama.2019.22176

11. Subirà C, Hernández G, Vázquez A, et al. Effect of Pressure Support vs T-Piece Ventilation Strategies During Spontaneous Breathing Trials on Successful Extubation Among Patients Receiving Mechanical Ventilation: A Randomized Clinical Trial. *JAMA*. 2019;321(22):2175-2182. doi:10.1001/jama.2019.7234

12. Thille AW, Muller G, Gacouin A, et al. Effect of Postextubation High-Flow Nasal Oxygen With Noninvasive Ventilation vs High-Flow Nasal Oxygen Alone on Reintubation Among Patients at High Risk of Extubation Failure: A Randomized Clinical Trial. *JAMA*. 2019;322(15):1465-1475. doi:10.1001/jama.2019.14901

13. Fowler AA 3rd, Truwit JD, Hite RD, et al. Effect of Vitamin C Infusion on Organ Failure and Biomarkers of Inflammation and Vascular Injury in Patients With Sepsis and Severe Acute Respiratory Failure: The CITRIS-ALI Randomized Clinical Trial. *JAMA*. 2019;322(13):1261-1270. doi:10.1001/jama.2019.11825

© 2025 Heirali A et al. *JAMA Network Open*.

14. Garrouste-Orgeas M, Flahault C, Vinatier I, et al. Effect of an ICU Diary on Posttraumatic Stress Disorder Symptoms Among Patients Receiving Mechanical Ventilation: A Randomized Clinical Trial. *JAMA*. 2019;322(3):229-239. doi:10.1001/jama.2019.9058

15. Vincent JL, Francois B, Zabolotskikh I, et al. Effect of a Recombinant Human Soluble Thrombomodulin on Mortality in Patients With Sepsis-Associated Coagulopathy: The SCARLET Randomized Clinical Trial. *JAMA*. 2019;321(20):1993-2002. doi:10.1001/jama.2019.5358

16. Hernández G, Ospina-Tascón GA, Damiani LP, et al. Effect of a Resuscitation Strategy Targeting Peripheral Perfusion Status vs Serum Lactate Levels on 28-Day Mortality Among Patients With Septic Shock: The ANDROMEDA-SHOCK Randomized Clinical Trial. *JAMA*. 2019;321(7):654-664. doi:10.1001/jama.2019.0071

17. Laterre PF, Berry SM, Blemings A, et al. Effect of Selepressin vs Placebo on Ventilator- and Vasopressor-Free Days in Patients With Septic Shock: The SEPSIS-ACT Randomized Clinical Trial. *JAMA*. 2019;322(15):1476-1485. doi:10.1001/jama.2019.14607

18. Russell DW, Casey JD, Gibbs KW, et al. Effect of Fluid Bolus Administration on Cardiovascular Collapse Among Critically Ill Patients Undergoing Tracheal Intubation: A Randomized Clinical Trial. *JAMA*. 2022;328(3):270-279. doi:10.1001/jama.2022.9792

19. Pickkers P, Mehta RL, Murray PT, et al. Effect of Human Recombinant Alkaline Phosphatase on 7-Day Creatinine Clearance in Patients With Sepsis-Associated Acute Kidney Injury: A Randomized Clinical Trial. *JAMA*. 2018;320(19):1998-2009. doi:10.1001/jama.2018.14283

20. Azoulay E, Lemiale V, Mokart D, et al. Effect of High-Flow Nasal Oxygen vs Standard Oxygen on 28-Day Mortality in Immunocompromised Patients With Acute Respiratory Failure: The HIGH Randomized Clinical Trial. *JAMA*. 2018;320(20):2099-2107. doi:10.1001/jama.2018.14282

21. Writing Group for the PReVENT Investigators, Simonis FD, Serpa Neto A, et al. Effect of a Low vs Intermediate Tidal Volume Strategy on Ventilator-Free Days in Intensive Care Unit Patients Without ARDS: A Randomized Clinical Trial. *JAMA*. 2018;320(18):1872-1880. doi:10.1001/jama.2018.14280

22. Perkins GD, Mistry D, Gates S, et al. Effect of Protocolized Weaning With Early Extubation to Noninvasive Ventilation vs Invasive Weaning on Time to Liberation From Mechanical Ventilation Among Patients With Respiratory Failure: The Breathe Randomized Clinical Trial. *JAMA*. 2018;320(18):1881-1888. doi:10.1001/jama.2018.13763

23. Dellinger RP, Bagshaw SM, Antonelli M, et al. Effect of Targeted Polymyxin B Hemoperfusion on 28-Day Mortality in Patients With Septic Shock and Elevated Endotoxin Level: The EUPHRATES Randomized Clinical Trial. *JAMA*. 2018;320(14):1455-1463. doi:10.1001/jama.2018.14618

24. Fossat G, Baudin F, Courtes L, et al. Effect of In-Bed Leg Cycling and Electrical Stimulation of the Quadriceps on Global Muscle Strength in Critically Ill Adults: A Randomized Clinical Trial. *JAMA*. 2018;320(4):368-378. doi:10.1001/jama.2018.9592

25. van Meenen DMP, van der Hoeven SM, Binnekade JM, et al. Effect of On-Demand vs Routine Nebulization of Acetylcysteine With Salbutamol on Ventilator-Free Days in Intensive Care Unit Patients Receiving Invasive Ventilation: A Randomized Clinical Trial. *JAMA*. 2018;319(10):993-1001. doi:10.1001/jama.2018.0949

26. van den Boogaard M, Slooter AJC, Brüggemann RJM, et al. Effect of Haloperidol on Survival Among Critically Ill Adults With a High Risk of Delirium: The REDUCE Randomized Clinical Trial. *JAMA*. 2018;319(7):680-690. doi:10.1001/jama.2018.0160

27. Frat JP, Quenot JP, Badie J, et al. Effect of High-Flow Nasal Cannula Oxygen vs Standard Oxygen Therapy on Mortality in Patients With Respiratory Failure Due to COVID-19: The SOHO-COVID Randomized Clinical Trial.

JAMA. 2022;328(12):1212-1222. doi:10.1001/jama.2022.15613

28. Alhazzani W, Parhar KKS, Weatherald J, et al. Effect of Awake Prone Positioning on Endotracheal Intubation in Patients With COVID-19 and Acute Respiratory Failure: A Randomized Clinical Trial. *JAMA*. 2022;327(21):2104-2113. doi:10.1001/jama.2022.7993

29. REMAP-CAP Writing Committee for the REMAP-CAP Investigators, Bradbury CA, Lawler PR, et al. Effect of Antiplatelet Therapy on Survival and Organ Support-Free Days in Critically Ill Patients With COVID-19: A Randomized Clinical Trial. *JAMA*. 2022;327(13):1247-1259. doi:10.1001/jama.2022.2910

30. Levy B, Girerd N, Amour J, et al. Effect of Moderate Hypothermia vs Normothermia on 30-Day Mortality in Patients With Cardiogenic Shock Receiving Venoarterial Extracorporeal Membrane Oxygenation: A Randomized Clinical Trial. *JAMA*. 2022;327(5):442-453. doi:10.1001/jama.2021.24776

31. Zampieri FG, Machado FR, Biondi RS, et al. Effect of Intravenous Fluid Treatment With a Balanced Solution vs 0.9% Saline Solution on Mortality in Critically Ill Patients: The BaSICS Randomized Clinical Trial. *JAMA*. 2021;326(9):1-12. doi:10.1001/jama.2021.11684

32. McNamee JJ, Gillies MA, Barrett NA, et al. Effect of Lower Tidal Volume Ventilation Facilitated by Extracorporeal Carbon Dioxide Removal vs Standard Care Ventilation on 90-Day Mortality in Patients With Acute Hypoxemic Respiratory Failure: The REST Randomized Clinical Trial. *JAMA*. 2021;326(11):1013-1023. doi:10.1001/jama.2021.13374

33. Driver BE, Semler MW, Self WH, et al. Effect of Use of a Bougie vs Endotracheal Tube With Stylet on Successful Intubation on the First Attempt Among Critically Ill Patients Undergoing Tracheal Intubation: A Randomized Clinical Trial. *JAMA*. 2021;326(24):2488-2497. doi:10.1001/jama.2021.22002

34. Ospina-Tascón GA, Calderón-Tapia LE, García AF, et al. Effect of High-Flow Oxygen Therapy vs Conventional Oxygen Therapy on Invasive Mechanical Ventilation and Clinical Recovery in Patients With Severe COVID-19: A Randomized Clinical Trial. *JAMA*. 2021;326(21):2161-2171. doi:10.1001/jama.2021.20714

35. Writing Committee for the REMAP-CAP Investigators, Estcourt LJ, Turgeon AF, et al. Effect of Convalescent Plasma on Organ Support-Free Days in Critically Ill Patients With COVID-19: A Randomized Clinical Trial. *JAMA*. 2021;326(17):1690-1702. doi:10.1001/jama.2021.18178

36. Zampieri FG, Machado FR, Biondi RS, et al. Effect of Slower vs Faster Intravenous Fluid Bolus Rates on Mortality in Critically Ill Patients: The BaSICS Randomized Clinical Trial. *JAMA*. 2021;326(9):830-838. doi:10.1001/jama.2021.11444

37. Johnstone J, Meade M, Lauzier F, et al. Effect of Probiotics on Incident Ventilator-Associated Pneumonia in Critically Ill Patients: A Randomized Clinical Trial. *JAMA*. 2021;326(11):1024-1033. doi:10.1001/jama.2021.13355

38. Gelissen H, de Grooth HJ, Smulders Y, et al. Effect of Low-Normal vs High-Normal Oxygenation Targets on Organ Dysfunction in Critically Ill Patients: A Randomized Clinical Trial. *JAMA*. 2021;326(10):940-948. doi:10.1001/jama.2021.13011

39. Roquilly A, Moyer JD, Huet O, et al. Effect of Continuous Infusion of Hypertonic Saline vs Standard Care on 6-Month Neurological Outcomes in Patients With Traumatic Brain Injury: The COBI Randomized Clinical Trial. *JAMA*. 2021;325(20):2056-2066. doi:10.1001/jama.2021.5561

40. Grieco DL, Menga LS, Cesarano M, et al. Effect of Helmet Noninvasive Ventilation vs High-Flow Nasal Oxygen on Days Free of Respiratory Support in Patients With COVID-19 and Moderate to Severe Hypoxemic Respiratory Failure: The HENIVOT Randomized Clinical Trial. *JAMA*. 2021;325(17):1731-1743. doi:10.1001/jama.2021.4682

© 2025 Heirali A et al. *JAMA Network Open*.

41. INSPIRATION Investigators, Sadeghipour P, Talasaz AH, et al. Effect of Intermediate-Dose vs Standard-Dose Prophylactic Anticoagulation on Thrombotic Events, Extracorporeal Membrane Oxygenation Treatment, or Mortality Among Patients With COVID-19 Admitted to the Intensive Care Unit: The INSPIRATION Randomized Clinical Trial. *JAMA*. 2021;325(16):1620-1630. doi:10.1001/jama.2021.4152

42. Meyhoff TS, Hjortrup PB, Wetterslev J, et al. Restriction of Intravenous Fluid in ICU Patients with Septic Shock. *N Engl J Med*. 2022;386(26):2459-2470. doi:10.1056/NEJMoa2202707

43. Hughes CG, Mailloux PT, Devlin JW, et al. Dexmedetomidine or Propofol for Sedation in Mechanically Ventilated Adults with Sepsis. *N Engl J Med*. 2021;384(15):1424-1436. doi:10.1056/NEJMoa2024922

44. Schjørring OL, Klitgaard TL, Perner A, et al. Lower or Higher Oxygenation Targets for Acute Hypoxemic Respiratory Failure. *N Engl J Med*. 2021;384(14):1301-1311. doi:10.1056/NEJMoa2032510

45. Hernández Martínez G, Rodriguez ML, Vaquero MC, et al. High-Flow Oxygen with Capping or Suctioning for Tracheostomy Decannulation. *N Engl J Med*. 2020;383(11):1009-1017. doi:10.1056/NEJMoa2010834

46. STARRT-AKI Investigators, Canadian Critical Care Trials Group, Australian and New Zealand Intensive Care Society Clinical Trials Group, et al. Timing of Initiation of Renal-Replacement Therapy in Acute Kidney Injury. *N Engl J Med*. 2020;383(3):240-251. doi:10.1056/NEJMoa2000741

47. Barrot L, Asfar P, Mauny F, et al. Liberal or Conservative Oxygen Therapy for Acute Respiratory Distress Syndrome. *N Engl J Med*. 2020;382(11):999-1008. doi:10.1056/NEJMoa1916431

48. Olsen HT, Nedergaard HK, Strøm T, et al. Nonsedation or Light Sedation in Critically Ill, Mechanically Ventilated Patients. *N Engl J Med*. 2020;382(12):1103-1111. doi:10.1056/NEJMoa1906759

49. ICU-ROX Investigators and the Australian and New Zealand Intensive Care Society Clinical Trials Group, Mackle D, Bellomo R, et al. Conservative Oxygen Therapy during Mechanical Ventilation in the ICU. *N Engl J Med*. 2020;382(11):989-998. doi:10.1056/NEJMoa1903297

50. National Heart, Lung, and Blood Institute PETAL Clinical Trials Network, Ginde AA, Brower RG, et al. Early High-Dose Vitamin D3 for Critically Ill, Vitamin D-Deficient Patients. *N Engl J Med*. 2019;381(26):2529-2540. doi:10.1056/NEJMoa1911124

51. François B, Cariou A, Clere-Jehl R, et al. Prevention of Early Ventilator-Associated Pneumonia after Cardiac Arrest. *N Engl J Med*. 2019;381(19):1831-1842. doi:10.1056/NEJMoa1812379

52. Lascarrou JB, Merdji H, Le Gouge A, et al. Targeted Temperature Management for Cardiac Arrest with Nonshockable Rhythm. *N Engl J Med*. 2019;381(24):2327-2337. doi:10.1056/NEJMoa1906661

53. Shehabi Y, Howe BD, Bellomo R, et al. Early Sedation with Dexmedetomidine in Critically Ill Patients. *N Engl J Med*. 2019;380(26):2506-2517. doi:10.1056/NEJMoa1904710

54. Arabi YM, Al-Hameed F, Burns KEA, et al. Adjunctive Intermittent Pneumatic Compression for Venous Thromboprophylaxis. *N Engl J Med*. 2019;380(14):1305-1315. doi:10.1056/NEJMoa1816150

55. Casey JD, Janz DR, Russell DW, et al. Bag-Mask Ventilation during Tracheal Intubation of Critically Ill Adults. *N Engl J Med*. 2019;380(9):811-821. doi:10.1056/NEJMoa1812405

56. Krag M, Marker S, Perner A, et al. Pantoprazole in Patients at Risk for Gastrointestinal Bleeding in the ICU. *N Engl J Med*. 2018;379(23):2199-2208. doi:10.1056/NEJMoa1714919

57. Girard TD, Exline MC, Carson SS, et al. Haloperidol and Ziprasidone for Treatment of Delirium in Critical Illness. *N Engl J Med*. 2018;379(26):2506-2516. doi:10.1056/NEJMoa1808217

© 2025 Heirali A et al. *JAMA Network Open*.

58. TARGET Investigators, for the ANZICS Clinical Trials Group, Chapman M, Peake SL, et al. Energy-Dense versus Routine Enteral Nutrition in the Critically Ill. *N Engl J Med*. 2018;379(19):1823-1834. doi:10.1056/NEJMoa1811687

59. Barbar SD, Clere-Jehl R, Bourredjem A, et al. Timing of Renal-Replacement Therapy in Patients with Acute Kidney Injury and Sepsis. *N Engl J Med*. 2018;379(15):1431-1442. doi:10.1056/NEJMoa1803213

60. Annane D, Renault A, Brun-Buisson C, et al. Hydrocortisone plus Fludrocortisone for Adults with Septic Shock. *N Engl J Med*. 2018;378(9):809-818. doi:10.1056/NEJMoa1705716

61. Venkatesh B, Finfer S, Cohen J, et al. Adjunctive Glucocorticoid Therapy in Patients with Septic Shock. *N Engl J Med*. 2018;378(9):797-808. doi:10.1056/NEJMoa1705835

62. Heyland DK, Wibbenmeyer L, Pollack JA, et al. A Randomized Trial of Enteral Glutamine for Treatment of Burn Injuries. *N Engl J Med*. 2022;387(11):1001-1010. doi:10.1056/NEJMoa2203364

63. Lamontagne F, Masse MH, Menard J, et al. Intravenous Vitamin C in Adults with Sepsis in the Intensive Care Unit. *N Engl J Med*. 2022;386(25):2387-2398. doi:10.1056/NEJMoa2200644

64. Finfer S, Micallef S, Hammond N, et al. Balanced Multielectrolyte Solution versus Saline in Critically Ill Adults. *N Engl J Med*. 2022;386(9):815-826. doi:10.1056/NEJMoa2114464

65. Ruijter BJ, Keijzer HM, Tjepkema-Cloostermans MC, et al. Treating Rhythmic and Periodic EEG Patterns in Comatose Survivors of Cardiac Arrest. *N Engl J Med*. 2022;386(8):724-734. doi:10.1056/NEJMoa2115998

66. REMAP-CAP Investigators, ACTIV-4a Investigators, ATTACC Investigators, et al. Therapeutic Anticoagulation with Heparin in Critically Ill Patients with Covid-19. *N Engl J Med*. 2021;385(9):777-789. doi:10.1056/NEJMoa2103417

67. Andersen-Ranberg NC, Poulsen LM, Perner A, et al. Haloperidol for the Treatment of Delirium in ICU Patients. *N Engl J Med*. 2022;387(26):2425-2435. doi:10.1056/NEJMoa2211868

68. TEAM Study Investigators and the ANZICS Clinical Trials Group, Hodgson CL, Bailey M, et al. Early Active Mobilization during Mechanical Ventilation in the ICU. *N Engl J Med*. 2022;387(19):1747-1758. doi:10.1056/NEJMoa2209083

69. REMAP-CAP Investigators, Gordon AC, Mouncey PR, et al. Interleukin-6 Receptor Antagonists in Critically Ill Patients with Covid-19. *N Engl J Med*. 2021;384(16):1491-1502. doi:10.1056/NEJMoa2100433

70. Cox CE, White DB, Hough CL, et al. Effects of a Personalized Web-Based Decision Aid for Surrogate Decision Makers of Patients With Prolonged Mechanical Ventilation: A Randomized Clinical Trial. *Ann Intern Med*. 2019;170(5):285-297. doi:10.7326/M18-2335

71. Heyland DK, Patel J, Compher C, et al. The effect of higher protein dosing in critically ill patients with high nutritional risk (EFFORT Protein): an international, multicentre, pragmatic, registry-based randomised trial. *Lancet*. 2023;401(10376):568-576. doi:10.1016/S0140-6736(22)02469-2

72. Jaber S, Paugam C, Futier E, et al. Sodium bicarbonate therapy for patients with severe metabolic acidaemia in the intensive care unit (BICAR-ICU): a multicentre, open-label, randomised controlled, phase 3 trial. *Lancet*. 2018;392(10141):31-40. doi:10.1016/S0140-6736(18)31080-8

73. Reignier J, Boisramé-Helms J, Brisard L, et al. Enteral versus parenteral early nutrition in ventilated adults with shock: a randomised, controlled, multicentre, open-label, parallel-group study (NUTRIREA-2). *Lancet*. 2018;391(10116):133-143. doi:10.1016/S0140-6736(17)32146-3

© 2025 Heirali A et al. *JAMA Network Open*.

74. INSPIRATION-S Investigators. Atorvastatin versus placebo in patients with covid-19 in intensive care: randomized controlled trial. *BMJ*. 2022;376:e068407. doi:10.1136/bmj-2021-068407

75. Gaudry S, Hajage D, Martin-Lefevre L, et al. Comparison of two delayed strategies for renal replacement therapy initiation for severe acute kidney injury (AKIKI 2): a multicentre, open-label, randomised, controlled trial. *Lancet*. 2021;397(10281):1293-1300. doi:10.1016/S0140-6736(21)00350-0

© 2025 Heirali A et al. *JAMA Network Open*.
