# Supplementary material for: Eligibility Criteria of Randomized Clinical Trials in Critical Care Medicine
Source: JAMA Netw Open. 2025 Jan 17;8(1):e2454944. doi: 10.1001/jamanetworkopen.2024.54944 (PMC11742542; doi:10.1001/jamanetworkopen.2024.54944)
Supplement: Supplement 2. — Canadian Critical Care Trials Group Members [file jamanetwopen-e2454944-s002.pdf]

Supplemental Online Content: Nonauthor Collaborators
*First name, last name, and suffix (if applicable) are required and will appear in PubMed.

*Group Name(s): Canadian Critical Care Trials Group							
***First Name and Middle Initial(s)**	***Last Name**	***Suffix (eg, Jr, III)**	Academic Degrees	Institution	Location (city, state/province, country)	Role or Contribution, eg, chair, principal investigator	Group (if more than 1 Group listed in the byline) and/or Subgroup (eg, Steering Committee)
Bram N.	Rochwerg		MD, MSc	McMaster University	Hamilton, ON, Canada	Manuscript Reviewer	Canadian Critical Care Trials Group Manuscript Committee Reviewer
Jennifer LY	Tsang		MD, PhD	McMaster University	Hamilton, ON, Canada	Manuscript Reviewer	Canadian Critical Care Trials Group Manuscript Committee Reviewer
