# Supplementary material for: Eligibility Criteria of Randomized Clinical Trials in Critical Care Medicine
Source: JAMA Netw Open. 2025 Jan 17;8(1):e2454944. doi: 10.1001/jamanetworkopen.2024.54944 (PMC11742542; doi:10.1001/jamanetworkopen.2024.54944)
Supplement: Supplement 3. — Data Sharing Statement [file jamanetwopen-e2454944-s003.pdf]

Data Sharing Statement

Heirali. Eligibility Criteria of Randomized Controlled Trials in Critical Care Medicine. *JAMA Netw Open*. Published January 17, 2025. doi:10.1001/jamanetworkopen.2024.54944

Data
Data available: Yes
Data types: Other (please specify), Data (not involving human participants)
Additional Information: Research participant data extracted from the included RCTs
How to access data: geeta.mehta@utoronto.ca
When available: With publication

Supporting Documents
Document types: None

Additional Information
Who can access the data: researchers whose proposed use of the data has been approved
Types of analyses: for the purpose specified in the approved proposal
Mechanisms of data availability: after approval of a proposal
